# Validation of Infant and Young Child Feeding Questionnaire for the Assessment of Knowledge, Attitudes and Practices among Child Care Providers: The IYCF-CCPQ

**DOI:** 10.3390/ijerph16122147

**Published:** 2019-06-17

**Authors:** Najihah Mahfuzah Zakria, Tengku Alina Tengku Ismail, Wan Nor Arifin Wan Mansor, Zaharah Sulaiman

**Affiliations:** 1 Department of Community Medicine, School of Medical Sciences, Universiti Sains Malaysia, Kubang Kerian, Kota Bharu, Kelantan 16150, Malaysia; drnajihahmahfuzah@gmail.com; 2 Unit of Biostatistics and Research Methodology, School of Medical Sciences, Universiti Sains Malaysia, Kubang Kerian, Kota Bharu, Kelantan 16150, Malaysia; wnarifin@usm.my; 3 Women’s Health Development Unit, School of Medical Sciences, Universiti Sains Malaysia, Kubang Kerian, Kota Bharu, Kelantan 16150, Malaysia; zaharah@usm.my

**Keywords:** infant young child feeding, questionnaire, validation, child care provider

## Abstract

The validation of a new questionnaire is essential to improving its credibility in the assessment and collection of evidence. This study aimed to validate a newly developed infant and young child feeding questionnaire for child care providers (IYCF-CCPQ) to measure the knowledge, attitudes, and practices regarding infant and young child feeding among them. A cross-sectional study was conducted with 200 child care providers who were involved in handling children less than two years old in child care centers in the northeastern part of Peninsular Malaysia. The IYCF-CCPQ was self-administered and consists of three domains: Knowledge (104 items), attitude (90 items), and practice (42 items). The dichotomous-scale items in the knowledge domain were analyzed using a two-parameter logistic model of item response theory (2-PL IRT). The Likert-type-scale items in the attitude section were assessed using exploratory factor analysis (EFA). The items in the practice section were assessed descriptively. Internal consistency by marginal reliability was assessed in the knowledge domain, and Cronbach’s alpha coefficient was used for the attitude domain. The marginal reliability values were 0.91 and 0.74 for the knowledge domains related to breastfeeding/formula feeding and complementary feeding, respectively, and the Cronbach’s alpha values were 0.89 and 0.90 for the attitude domains related to breastfeeding/formula feeding and complementary feeding, respectively. The analysis showed good psychometric properties (discrimination, difficulty index, factor loading, and communalities) and good reliability. The IYCF-CCPQ is valid for use assessing the knowledge, attitudes, and practices among Malaysian child care providers regarding infant and young child feeding.

## 1. Introduction

Adequate nutrition is critical to child health and development. It is well recognized that the period from birth to two years of age is a “critical window” for the promotion of optimal growth, health, and behavioral development [1]. With increasing participation rates of women in the workforce, providing nutritional support in the child care setting has become crucial, as child care providers act as the front line in taking care of children [2,3]. To ensure the availability and sustainability of infant and young child feeding-friendly environments, there is a need to identify the knowledge, attitudes, and practices regarding infant and young child feeding among child care providers and to upskill providers as necessary. 

To our knowledge, validated questionnaires specifically to assess child care providers’ knowledge, attitudes, and practices regarding infant and young child feeding in Malaysia in detail are not yet available. The lack of such questionnaires is in contrast to the many available questionnaires that focus more on the mother and health care provider. Furthermore, the locally available questionnaire focused on breastfeeding rather than infant and young child feeding comprehensively. On the other hand, many studies [4,5,6] in various populations from western countries that focus on the assessment of child care providers regarding infant and young child feeding were shown to utilize questionnaires that were not properly validated and measured a different concept. As study by Lucas in United States, focused on the assessment of child care providers’ knowledge and attitude regarding the support of breastfeeding in United States. The knowledge aspect covered the advantages, practicality, maternal condition, and national policy around breastfeeding. Meanwhile, the attitude aspect covered the cognitive and affective components of breastfeeding. However, the limitation of the study was the unvalidated attitude and knowledge scales, as well as weak internal consistency and reliability for the knowledge component [4].

There is also a questionnaire from Freedman and Alvarez, which covers the assessment of knowledge, attitudes, and practices regarding early childhood feeding among child care providers. This questionnaire was modified from the Stanford Child Feeding Questionnaire [7] and the Hughes Caregiver Feeding Styles Questionnaire [8]. The attitude component of the questionnaire covered cognitive and affective components, not behavioral components. The practice component covered responsive feeding and the practical aspects of infant and young child feeding. However, the limitation of this study was its instrument validity [5]. A study by Clark in Colorado, also produced a questionnaire assessing child care providers’ knowledge, attitudes, behavior, and training related to infant feeding, specifically breastfeeding. The instrument validity was only measured up to the pretesting of the questionnaire [6]. Meanwhile, a newly developed questionnaire from Australia named Feeding Practices and Structure Questionnaire (FPSQ) provides a new reliable and valid measure of parental feeding practices; however, it only focuses on complementary feeding [9].

In view of the unavailability of questionnaires covering the whole infant and young child feeding concept [4,5,6,9], it is important to develop a valid and reliable questionnaire that is best suited to the local culture, belief, and practices in order to provide useful and comparable data about infant and young child feeding among child care providers in Malaysia. 

Validity and reliability studies are essential to increase the credibility of the questionnaire as a research tool that can produce valid data. This also helps collect good-quality data with high comparability and increase the credibility of the data for generalization to the population. This study aims to determine the validity and reliability of a newly developed infant and young child feeding questionnaire for the assessment of the knowledge, attitudes, and practices among child care providers in Kota Bharu, Kelantan, Malaysia.

## 2. Materials and Methods 

### 2.1. Study Design and Participants

A cross-sectional study was conducted from April 2017 to September 2017 to explore the psychometric properties of the questionnaire. This study evaluated 200 child care providers from 53 registered child care centers in six districts in Kelantan. Purposive sampling was applied for the recruitment of the respondents since there were a limited number of registered child care providers. All child care providers with a minimum of six months working experience and who had ever experienced caring for children less than two years old in child care centers were invited to participate in this study. A combination of respondents with different age categories, durations of work, and marital statuses were included as participants.

### 2.2. Research Tool

A newly developed questionnaire called the infant and young child feeding questionnaire for child care providers (IYCF-CCPQ) was used as the research tool for this study. This questionnaire was developed in November 2016 and completed in February 2017. The questionnaire was written in the Malay language and is applicable to the child care providers in Kelantan. It was designed to be self-administered, with an estimated time to complete the questionnaire based on the pretesting phase of 45 min.

#### 2.2.1. Development of the IYCF-CCPQ Questionnaire

Development of the questionnaire started with an item generation and conceptualization process, followed by cognitive debriefing and pre-testing as part of the response process assessment. The IYCF-CCPQ was structured and designed specifically for the assessment of knowledge, attitudes, and practices regarding infant and young child feeding among child care providers. In general, Delphi technique was used to maintain the anonymity and confidentiality of input between experts as there were facilitator that coordinate and gained the input from all experts. However, the credibility of the technique was argued as initial content is determined by a lead investigator that lead to the biased toward selection of items by the investigator. Thus, modified Delphi techniques were used in the development of this questionnaire [10]. This technique involved more diverse panel of experts in which an international certified lactation consultant, two public health physicians, a biostatistician, a nutritionist, a social welfare officer, and a representative from non-governmental organization that related to child care providers. 

Item generation was based on discussions with experts and from a literature review. There were four experts involved who also acted as research team members. They consist of an international certified lactation consultant, two public health physicians, and a biostatistician. This group of experts was purposely selected based on their expertise on and experience with the measured concepts in the newly developed questionnaire. 

An extensive literature review on questionnaire development was conducted by the research team members. A literature search was done regarding infant and young child feeding, specifically focusing on breastfeeding, complementary feeding, and formula feeding, the role of child care providers, the assessment of knowledge, attitudes, and practices (KAP), available questionnaires [4,5,6,9], child care services and infant and young child feeding-friendly center. Key words used in the database searches were “infant and young child feeding”, “child care providers”, “breastfeeding”, “complementary feeding”, “KAP” and “child care centre”. Databases used included Cochrane Library, PubMed, Medline (Web science), Scopus, and Medline Ovid. Every research team member reviewed comprehensively a number of questionnaires that differed markedly in term of domains and theoretical backgrounds, as well as in their validation approaches and the quality of the validation evidence. The research team also reviewed various local and internationally published infant and young child feeding guidelines, as well as training guidelines for child care providers [11,12,13,14,15,16,17]. Relevant literature, including quantitative and qualitative studies as well as relevant theoretical frameworks [18], were utilized to incorporate more ideas and assist the questionnaire development. 

The theoretical background of the tri-partite theory was used for the attitude domain, which involved evaluations of people, objects, and ideas [18]. The theory highlighted three main components for attitude assessment—which were cognitive, affective, and behavioral—that combine to form an overall evaluation of the attitude object. The measuring component for cognitive, affective, and behavior is described in Table 1.

Every research team member suggested possible domains based on their own experience and literature reviews. Each contributed domain was continuously appraised until all members agreed to focus on a number of identified domains. 

Relevant and representative items covering both positively and negatively worded items were identified. At least five items per component were identified to cover representativeness, relevancy, and consistency with the intended meaning of the construct. Verified domains were defined within the context of the assessment of infant and young child feeding among child care providers.

The newly developed questionnaire (IYCF-CCPQ) contained 236 items representing the three domains: Knowledge, attitude, and practice. The knowledge and attitude domains of the IYCF-CCPQ each have two sections: Section A for breastfeeding and formula feeding and section B for complementary feeding. The knowledge domain contained 104 items separated into section A (67 items for the assessment of breastfeeding and formula feeding: KA1 to KA67) and section B (37 items for the assessment of complementary feeding: KB1 to KB37). The attitude domain comprised 90 items separated into section A (40 items for the assessment of breastfeeding and formula feeding: AA1 to AA40) and section B (50 items for the assessment of complementary feeding: AB1 to AB50). The remaining 42 items belonged to the practice domain, which covered six sub-sections: Handling express breastmilk, giving express breastmilk, handling formula milk, handling a feeding bottle, food storage, and responsive feeding. These sections assessed the practical handling of breastfeeding, formula feeding, and complementary feeding. 

#### 2.2.2. Response Process: Cognitive Debriefing and Pre-Testing

After the development of the questionnaire, the response processes of the questionnaire were assessed. Cognitive debriefing and pretesting were done. Cognitive debriefing was conducted among eight child care providers from one registered child care center in Kota Bharu, one nutritionist from Kelantan Health State Department and one representative from a non-governmental organization (NGO). This NGO was related to child care providers and child care centers (PERASCO). Cognitive debriefing was done using the methods of think-aloud and verbal probing. The understandability of the questionnaire and errors that may be introduced into the questionnaire were assessed, which involved interpreting specific questions, recalling necessary information, performing judgments, and editing answers. This stage was able to highlight any items that may have been inappropriate at a conceptual level and to identify any other issues that confused the respondents [19]. 

This was followed by a pre-testing phase in which an assessment was done to evaluate the questionnaire, including its overall flow (including transitions between sections), the length of the questionnaire, the level of respondent interest and attention, whether it was user friendly for the respondent, how well the respondents understood and answered the questions correctly, and the maximum time required to answer the entire questionnaire [20]. 

Pretesting was done with 30 child care providers from nine registered child care centers in Pasir Mas, Kelantan. A review of the pre-testing results and finalization was performed to incorporate the findings of the pre-testing process to improve the quality of the questionnaire in comparison with the original version. Respondents’ interpretations of the items and discrepancies among these were highlighted. The results of the pre-testing were explained and discussed with the research team members. The members evaluated all the comments and suggestions given by respondents, and necessary amendments were made accordingly. During the pre-testing, the comments in general and acceptance of the questionnaire were good, although a long time was required to answer the whole questionnaire. The overall mean time required for respondents to answer all the items was 45.5 (SD 10.45) minutes. Revision of the questionnaire was done accordingly following the pre-testing phase, and 236 items remained for the internal structure validity assessment.

#### 2.2.3. Scoring Method and Response Options

The items in the knowledge domain had three options of “true”, “false”, and “don’t know”. One point was given for a correct answer and zero points given for an incorrect or “don’t know” answer. Thus, the possible score of this domain ranged from 0 to 67 for section A and 0 to 37 for section B. The scores for each section were calculated. They were then converted to percentage scores by dividing by the possible maximum score and multiplying by 100. A higher percentage score indicated better knowledge of the items tested.

The items in the attitude domain were scored on a five-point Likert scale from one (strongly disagree) to five (strongly agree). Points were given in ascending order as follows: 1 = “strongly disagree”, 2 = “disagree”, 3 = “unsure”, 4 = “agree”, and 5 = “strongly agree”. The positive and negative statements were arranged randomly throughout the questionnaire to avoid habitual bias from the respondents.

The score contribution for the positive statements was the scale position and the contribution for the negative statements was reverse score. The scores in this domain can range from 40 to 200 for section A and 50 to 250 for section B. The practice domain contained 42 items that were rated on a four-point Likert scale. Points were given on ascending order as follows: 1 = “never”, 2 = “seldom”, 3= “sometimes”, and 4 = “always”. Thus, each item in the practice domain had four-Likert scale responses ranging from one to four with one representing “never” and four representing “always”. Each item in the practice domain was reported descriptively.

### 2.3. Method of Data Collection

The respondents were first briefed about the study. Informed consent was then obtained from the respondents who agreed to be involved in the study. The IYCF-CCPQ questionnaire (in Supplementary Materials) forms were given to each participant for self-administration. The complete forms were collected on the same day to reduce information bias. The respondents were asked to choose one best response for each statement in the questionnaire, and ample time was given to the respondents to answer all the questions.

### 2.4. Study Sample Size

A study sample size of 150 is required for an exploratory factor analysis (EFA) whenever 10 or more items are expected to have factor loadings of 0.4 [21,22]. Given this, the required sample size for the two-parameter logistic model of item response theory (2-PL IRT) of at least 200 is adequate for analysis [23]. The reliability of the construct (Cronbach alpha) was calculated based on a 0.05 significance level, the power of 0.80, 25 items, an acceptable alpha of 0.7, and an expected Cronbach alpha of 0.80 with no drop-out encounter in this sample size estimation. Thus, the highest sample size yielded of 200 child care providers was sufficient for this study to determine validity of the new questionnaire.

### 2.5. Statistical Method

The psychometric assessment involved EFA and item response theory (IRT) analysis. Data analysis was computed using R software version 3.3.4 and the R studio environment [24]. The dichotomous scale items of the knowledge domain were analyzed using 2-PL IRT using the *ltm* package. A difficulty index in the range of −3 to +3 and discrimination index in the range of 0.35 to 2.5 were considered acceptable [25,26,27]. Item fit was determined by the chi-square goodness-of-fit per item [26], and unidimensionality was determined using modified parallel analysis [28]. The polytomous scale items and hypothetical concept of items in the attitude domain were assessed using EFA. The principal axis factoring extraction method with oblimin rotation was applied in the EFA. To determine the number of extracted factors, eigenvalues > 1.0, parallel analysis, and scree plot inspection were performed [29]. Factor loadings of a minimum of 0.3 were considered acceptable [22]. Practice domain items were validated descriptively per item by presenting the count and percentage.

For reliability analysis, the internal consistency according to marginal reliability was used in the IRT because the marginal reliability can be used to estimate the average reliability of the respondent’s knowledge. The exact value of acceptable marginal reliability suggested that a value of 0.623 is acceptable [30]. Meanwhile, a Cronbach’s alpha coefficient ≥ 0.70 was considered an adequate internal consistency reliability for polytomous scale-items [31].

### 2.6. Ethical Consideration

This research was registered with National Medical Research Registration (NMRR) on October 19, 2016 (NMRR ID Number: NMRR-16-1837-32901). Ethical clearance approval was obtained from the Research Ethics Committee (Human), *Universiti Sains Malaysia* (JEPeM code: USM/JEPeM/16100405) on January 25, 2017. Approval from the Department of Social Welfare, Putrajaya (Reference no.: JKMM 100/12/5/2:2017/061) was obtained on March 10, 2017. The confidentiality of the data has been strictly maintained. Only the authors had access to the data.

## 3. Results

### 3.1. Socio-Demographic Characteristics of Child Care Providers

The majority of child care providers involved in this study were female, single, and young, with a mean age of 31.1 (SD = 10.29) years. The majority (169, 84.5%) of the respondents had less than a diploma in terms of academic qualification. Their median total working experience was 3.0 (IQR = 4.50) years. Table 2 further details the socio-demographic characteristics of the child care providers.

### 3.2. Item Response Theory

#### 3.2.1. Knowledge (Breastfeeding and Formula Feeding Section)

Based on a 2-PL IRT assessment in the knowledge section, item KA3 had a low difficulty estimate of −9.2, while item KA31 had an extreme difficulty estimate of 10.01. KA62 had a negative discrimination estimate of −0.3. These items were subsequently removed. The IRT analysis of the remaining items is summarized in Table 3.

As shown by the IRT analysis, the psychometric properties of the knowledge domain (breastfeeding and formula feeding section) were good. With regard to the difficulty parameter, all the knowledge items were within or close to the acceptable range of –3 to +3, and they ranged from –5.6 to +1.7. The KA14 item slightly exceeded the cut-off value. However, in accordance with the advice of the experts, the item was retained because the content of this item was important. In terms of discrimination, most of the items were within the acceptable range of 0.35 to 2.5, and they ranged from 0.3 to 2.4.

The item goodness-of-fit showed that 13 of the items did not fit well (*p* < 0.05). However, all these items were also retained in this section because they had acceptable difficulty and discrimination values. The amount of total information trapped by the items between the -3 to +3 ranges of ability was 83.21%. Internal consistency by marginal reliability was 0.91.

There were ultimately 64 items retained in the final model of the knowledge domain (breastfeeding and formula feeding section). The mean score for knowledge was 67.19% (SD = 15.64%).

#### 3.2.2. Knowledge (Complementary Feeding Section)

Based on a 2-PL IRT assessment on the knowledge section, item KB33 had a very low difficulty and a negative discrimination estimate of −51.19 and −0.03, respectively. In addition, item KB35 had a low discrimination estimate of 0.22 and exceeded the upper limit of the difficulty cut-off value, with an estimate of 3.66. These items were subsequently removed. The IRT analysis of the remaining items is summarized in Table 4.

As shown by the IRT analysis, the psychometric properties of the knowledge domain (complementary feeding section) were in the acceptable range. With regard to the difficulty parameter, all the knowledge items were within or close to the acceptable range of -3 to +3, with KB8, KB12, KB14, KB17, KB19, and KB21 slightly exceeding the cut-off value. However, in accordance with the advice of the experts, these items were retained because of their importance. Items KB8, KB12, KB14, KB17, and KB19 related to the type of food that is suitable to be given to children, while item KB21 concerned whether infants at the age of nine months can be allowed to feed by themselves; all these items were not measured by other items in the questionnaire. In terms of discrimination, most of the items were within the acceptable range of 0.35 to 2.5, and they ranged from 0.05 to 3.2.

The item goodness-of-fit showed that nine of the items did not fit well (*p* < 0.05). However, all these items were also retained in this section because they had acceptable difficulty and discrimination values. The amount of total information trapped by the items between −3 and +3 ranges of ability was 77.3%. The internal consistency by marginal reliability was 0.74.

There were ultimately 35 items retained in the final model of the knowledge domain (complementary feeding section). The mean score for knowledge was 68.72 (SD = 12.54).

### 3.3. Exploratory Factor Analysis

#### 3.3.1. Attitude (Breastfeeding and Formula Feeding Section)

For the attitude domain, the data correlation matrix was factorable, and the assumptions needed to conduct EFA were met, as indicated by a Kaiser–Meyer Olkin (KMO) value of 0.77 and Bartlett’s test of sphericity being significant (*p* < 0.05). This attitude domain was designed using the tri-partite theory [18], which consists of the affective, behavioral, and cognitive components relating to breastfeeding. However, based on the eigenvalue, observation of the scree plot and the cumulative percentage of variance, only one factor solution was determined.

All the items in the attitude domain that had an acceptable factor loading range between 0.3 and 0.67 were retained. AAA23, AAA26, AAA28, AAB33, AAC13, and AAC17 had low factor loading and were removed. Table 5 details the value of the factor analysis.

This attitude factor with a reduced number of items had good reliability, as indicated by a Cronbach’s alpha of 0.89, and had good content coverage in relation to the attitude concept. There were ultimately 34 items retained in the final model of attitude (breastfeeding and formula feeding section). The mean score for attitude (breastfeeding and formula feeding section) was 158.71 (SD = 14.32).

#### 3.3.2. Attitude (Complementary Feeding Section)

For this section, the data correlation matrix was factorable, and the assumptions needed to conduct EFA were met, as indicated by a KMO value of 0.78 and Bartlett’s test of sphericity being significant (*p* < 0.05). This attitude domain was designed using the tri-partite theory of Lawrence [18], which consists of affective, behavioral, and cognitive components relating to complementary feeding. However, based on the eigenvalue, observation of the scree plot and the cumulative percentage of variance, only one factor solution was determined.

All the items in the attitude domain that had an acceptable factor loading range between 0.3 and 0.71 were retained. ABA26, ABA29, ABB47, and ABC15 had low factor loading and were removed. ABB44, ABC19, and ABC21 had negative factor loading and were removed based on the experts’ discussion. Table 6 details the value of the factor analysis.

This attitude factor with a reduced number of items had good reliability, as indicated by a Cronbach’s alpha of 0.90, and had good content coverage in relation to the attitude concept. There were ultimately 43 items retained in the final model of attitude (complementary feeding section). The mean score for attitude (complementary feeding section) was 191.97 (SD = 15.77).

### 3.4. Practice

Items in the practice domain were validated by content and described descriptively per each item by count and percentage, as in Table 7.

### 3.5. Final Validated IYCF-CCPQ

Table 8 summarizes the items in each of the sections of the IYCF-CCPQ before and after psychometric analysis. The final validated questionnaire (IYCF-CCPQ) stands for ‘*Borang Kaji Selidik Pemakanan Bayi dan Kanak-kanak dalam kalangan Pengasuh*’. The validated questionnaire has a total of 218 items in three major domains: knowledge, attitude, and practice.

## 4. Discussion

The main aim of this study was to validate a new IYCF-CCPQ questionnaire on infant and young child feeding in Malaysia, specifically in Kelantan. Overall, the questionnaire was intended to assess the knowledge, attitudes, and practices among child care providers in registered child care centers. The majority of the respondents were Malays (98.5%) who were native speakers of the Malay language. This finding was consistent with the Kelantan population, as there the Malay ethnicity is the majority 96% of the state [32]. This met the purpose of this study to validate new tools that have been adapted culturally.

The dichotomous scale items in the knowledge domain used IRT, while the polytomous scale items of the attitude domain were validated using EFA. IRT was beneficial because it was able to discriminate between respondents with good and poor knowledge, determine the difficulty of the questionnaire and determine good and poor items for the questionnaire. EFA is essential to searching the latent constructs of the items and thereby allowing some theory to be formulated. Using EFA, the number of factors and quality of the items can be assessed. Common factors extracted and grouped from the list of the items and the relationships among them can be determined. After regrouping, the naming of the extracted factor is essential to reduce the variable complexity for greater simplicity [33].

Generally, the knowledge domain showed good psychometric properties based on the difficulty and discriminatory parameters of the items. However, some items had to be removed from the knowledge domain of IYCF-CCPQ due to poor discrimination and a high difficulty parameter. Poor discrimination indicates that the items are unable to discriminate between high-scoring respondents and low-scoring respondents. A high difficulty index reflects the poor ability of the respondents to answer the item. Only relevant items were retained in order to differentiate between high- and low-knowledge respondents.

The validation study of the attitude domain revealed a good-fitting one-factor model of attitude (affective-behavioral-cognitive) instead of the proposed three-factor model based on the tri-partite theory consisting of affective, behavioral, and cognitive elements [18]. Using only one factor minimized items overlapping and obtained better factor loading. Furthermore, this will improve the final outcome of the questionnaire through its good validity and reliability. Based on the EFA results, the attitude domain had good construct validity and reliability.

The factor analytic approach was unsuitable for the practice domain due to the absence of interpretable correlations between the items. Thus, the scores for each item were utilized rather than the total scores for the domain. This concept was applied similarly to another study [34]. In this study, a descriptive explanation of the type of practice was required for each item. These items reflected what the expert panel considered important to infant and young child feeding practices in the community. Understanding practices that are lacking among the assessed child care providers is important to better plan effective intervention strategies. Thus, all items assessing the practice domain were maintained, as the content was important and relevant to the context.

The conventional way to interpret reliability using Cronbach’s alpha is not meaningful in an IRT analysis of the knowledge items because of its dichotomous type of questions, as opposed to polytomous type in the attitude and practice domains [30]. The marginal reliability can be used to estimate the average reliability of the respondent’s knowledge. The exact value of acceptable marginal reliability is not well documented, but we based our statistical analysis on studies by Dimitrov [30] that suggested that a value of 0.623 was acceptable. Another point to note is that marginal reliability will be influenced by the removal of some items [30]. In this study, the removal of a few items that had extreme results significantly improved the marginal reliability score of the knowledge items. The good reliability of the attitude domain indicated the consistency and homogeneity of the items in the domain [35].

This study had several strengths. First, to the best of the researchers’ knowledge, the IYCF-CCPQ questionnaire was tailored the cultural background of the respondents and provided better information specific to child care providers in Malaysia, especially among the Malay ethnic group. Thus, this validated new questionnaire will be useful, and the baseline result can be used in the future to implement an intervention program for child care providers that could potentially benefit a large proportion of the Malaysian population.

A limitation of this study is it was confined to the child care providers in registered child care centers in Kelantan, which represent only the north-eastern part of Peninsular Malaysia. Cross-validation studies are needed in other parts of Peninsular Malaysia, as well as western and eastern Malaysia, that involve other ethnicities and other child care centers in order to determine the validity and reliability of the IYCF-CCPQ for a wider population. Furthermore, future studies can be conducted to substantiate the theory generated by this EFA result through confirmatory factor analysis.

## 5. Conclusions

The IYCF-CCPQ has been shown to have good psychometric properties. It is a valid and reliable instrument to evaluate the knowledge, attitudes, and practices among child care providers regarding infant and young child feeding. The questionnaire consisted of 218 items (99 items on knowledge, 77 items on attitude, and 42 items on practice). The knowledge, attitude, and practice domains were psychometrically valid according to IRT and factor analytic and descriptive evidence.

## 6. Patents

The IYCF-CCPQ has been registered as intellectual property with MyIPO, and its copyright is held by Universiti Sains Malaysia (Application number: LY2018003000).

## Figures and Tables

**Table 1:** Tri-partite theory of attitude.

Component	Description
Cognitive	Thoughts or beliefs about attitude object that involve: (a) Cognitive dissonance theory: state of emotional discomfort when holding contradictory beliefs or when beliefs contradict their behavior (b) Self-perception theory: uncertain of their attitudes; attitudes inferred by observing their own behavior
Affective	Emotion or feeling towards attitude object
Behavior	Action or behavior towards attitude object

**Table 2:** Sociodemographic characteristic of child care providers (n = 200).

Variables	Mean (SD)	*n* (%)
Age (year)	31.1 (10.29)	
Gender (female)		200 (100.0)
Ethnicity		
Malay		197 (98.5)
Chinese		3 (1.5)
Education level		
Less than a diploma		169 (84.5)
Diploma and higher		31 (15.5)
Marital status		
Married		92 (42.6)
Single/unmarried		99 (49.5)
Employment		
Total working experience (years)	3.0 (4.50) *	
Experience in current centers (years)	2.3 (4.10) *	
Total working hours per day	10.04 (1.22)	
Workload		
Total children in center	22.8 (9.93)	
Total children cared per provider	10.4 (7.41)	
Total provider per center	4.5 (1.65)	
Employment scope **		
Care of child under 2 y/o		136 (68.0)
Care of child more than 2 y/o		170 (85.0)
Give food as scheduled		174 (87.0)
Serve food		128 (64.0)
Monitor child during eating		171 (85.5)
Operation hours (center)		
Overtime service	10.6 (0.86)	100 (50.0)
Involvement with overtime service		84 (42.0)
Self-experience		
Had own-child		84 (42.0)
Total children (*n* = 84)	1.3 (1.79)	
Had breastfeeding experience		79 (39.5)
Maximum breastfeeding duration (*n* = 79) (month)	8.38 (11.24)	
Exclusive breast feeding for 6 month (yes)		38 (19.0)
Age of starting complementary food (*n* = 84) (month)	2.88 (3.95)	
Facilities at childcare centers **		
Mother is able to breastfeed her child in the childcare center		142 (71.0)
Breastfeeding corner		96 (48.0)
Refrigerator for EBM		157 (78.5)
Breast pump		13 (6.5)
Educational material		83 (41.5)
Training		
KAKP course (involvement)		97 (48.5)
Last training (years) (*n* = 97)		
Other relevant course	1.85 (2.59)	83 (41.5)
Information system		
Own initiative to search for information		105 (52.5)
The frequency of searching information (per month)	1.40 (2.03)	
Source **		
Internet		95 (47.5)
Books		75 (37.5)
Pamphlet		42 (21.0)
Magazines		59 (29.5)
Support group		9 (4.5)
Health care provider		38 (19.0)
Non-governmental organization		7 (3.5)
Information source category		
No source		90 (40.5)
At least one source information		22 (11.0)
More than one source information		88 (44.0)

*median (IQR), **respondents may answer more than one options, KAKP = basic childcare course.

**Table 3:** Item response theory parameters estimate of items of knowledge in breastfeeding and formula feeding section of infant and young child feeding questionnaire for child care providers (IYCF-CCPQ).

Item Parameters			S-X^2^ Fit Index			
Item after Removal	Difficulty (SE)	Discrimination (SE)	Standardized Loading	X^2^	df	*p*-Value
KA1	−0.304 (0.099)	2.415 (0.433)	0.817	18.936	17	0.332
KA2	−2.561(0.534)	1.429 (0.390)	0.643	3.761	6	0.709
KA4	−1.213 (0.250)	1.208 (0.262)	0.579	22.919	19	0.241
KA5	0.186 (0.158)	1.080 (0.224)	0.536	29.079	23	0.178
KA6	−1.664 (0.274)	1.886 (0.423)	0.742	11.289	10	0.335
KA7	−1.016 (0.183)	1.604 (0.341)	0.686	39.828	18	**0.002**
KA8	−2.466 (0.613)	0.898 (0.264)	0.467	24.011	15	0.065
KA9	−2.553 (0.609)	0.912 (0.274)	0.472	26.484	14	**0.022**
KA10	−0.557 (0.139)	1.588 (0.315)	0.682	25.823	20	0.172
KA11	−1.099 (0.278)	0.982 (0.222)	0.500	17.396	23	0.789
KA12	−0.901 (0.258)	0.868 (0.210)	0.454	19.777	24	0.709
KA13	−3.320 (0.839)	1.137 (0.376)	0.556	4.173	4	0.383
KA14	−5.674 (2.581)	0.473 (0.274)	0.268	17.486	9	**0.042**
KA15	−2.119 (0.397)	1.685 (0.446)	0.703	7.355	7	0.393
KA16	−1.884 (0.362)	1.450 (0.346)	0.649	11.224	12	0.510
KA17	0.377 (0.113)	1.926 (0.360)	0.749	21.028	15	0.136
KA18	−1.573 (0.283)	1.559 (0.334)	0.675	10.683	12	0.556
KA19	−0.128 (0.094)	2.432 (0.440)	0.819	21.145	17	0.220
KA20	−0.917 (0.163)	1.775 (0.342)	0.722	20.219	18	0.321
KA21	−1.689 (0.389)	0.926 (0.243)	0.478	34.139	21	**0.035**
KA22	−1.872 (0.540)	0.728 (0.207)	0.393	30.636	23	0.132
KA23	−1.161 (0.274)	1.013 (0.231)	0.511	30.074	23	0.147
KA24	−0.748 (0.206)	1.063 (0.237)	0.530	34.274	23	0.061
KA25	0.378 (0.106)	2.089 (0.390)	0.775	17.281	15	0.302
KA26	−0.838 (0.178)	1.527 (0.308)	0.668	36.379	20	**0.014**
KA27	−0.013 (0.093)	2.393 (0.446)	0.815	18.579	16	0.291
KA28	−1.438 (0.379)	0.795 (0.210)	0.817	18.999	19	0.263
KA29	1.200 (0.335)	0.826 (0.209)	0.437	22.445	19	0.263
KA30	−2.398 (0.524)	1.169 (0.319)	0.566	13.552	10	0.194
KA32	−0.486 (0.167)	1.193 (0.239)	0.574	17.763	23	0.770
KA33	−3.961 (1.513)	0.759 (0.294)	0.407	5.849	7	0.557
KA34	−0.769 (0.202)	1.070 (0.242)	0.532	22.782	23	0.474
KA35	−1.909 (0.473)	0.862 (0.236)	0.452	35.901	20	**0.016**
KA36	−0.013 (0.118)	1.544 (0.298)	0.672	29.398	21	0.105
KA37	−0.177 (0.185)	0.889 (0.205)	0.463	42.498	24	**0.011**
KA38	0.146 (0.118)	1.586 (0.293)	0.682	36.271	19	**0.010**
KA39	−1.260 (0.241)	1.455 (0.304)	0.650	20.728	17	0.239
KA40	1.559 (0.342)	1.037 (0.247)	0.520	30.133	17	**0.025**
KA41	−0.516 (0.167)	1.168 (0.241)	0.566	28.525	23	0.197
KA42	−0.405 (0.203)	0.843 (0.208)	0.444	17.965	24	0.805
KA43	0.156 (0.265)	0.563 (0.176)	0.314	31.593	25	0.170
KA44	−0.066 (0.162)	0.997 (0.221)	0.505	33.546	23	0.072
KA45	−1.173 (0.294)	0.965 (0.224)	0.493	41.072	23	**0.012**
KA46	−3.989 (1.748)	0.525 (0.228)	0.295	22.104	14	0.077
KA47	1.625 (0.474)	0.649 (0.202)	0.357	19.825	20	0.469
KA48	−0.496 (0.179)	1.014 (0.225)	0.512	28.361	24	0.245
KA49	−0.705 (0.208)	0.997 (0.224)	0.506	20.226	24	0.684
KA50	−2.291 (0.454)	1.475 (0.387)	0.655	3.247	7	0.861
KA51	−0.051 (0.190)	0.811 (0.202)	0.430	34.704	24	0.073
KA52	0.076 (0.164)	1.014 (0.215)	0.512	26.848	23	0.262
KA53	−0.670 (0.244)	0.869 (0.199)	0.455	21.717	24	0.596
KA54	−1.465 (0.767)	0.359 (0.163)	0.206	22.240	26	0.675
KA55	−3.068 (0.751)	1.146 (0.361)	0.559	4.778	5	0.444
KA56	0.450 (0.302)	0.525 (0.175)	0.295	33.533	26	0.147
KA57	−0.178 (0.180)	0.881 (0.209)	0.460	35.554	25	0.079
KA58	−1.425 (0.322)	1.028 (0.245)	0.517	16.503	21	0.741
KA59	1.719 (1.567)	0.177 (0.154)	0.104	39.868	27	0.053
KA60	−1.865 (0.852)	0.393 (0.169)	0.225	35.485	26	0.102
KA61	−1.501 (0.276)	1.455 (0.321)	0.650	18.947	13	0.125
KA63	−1.404 (0.914)	0.295 (0.158)	0.171	39.272	26	**0.046**
KA64	−3.236 (1.058)	0.555 (0.219)	0.310	31.032	19	**0.040**
KA65	−2.184 (0.683)	0.624 (0.202)	0.344	35.754	23	**0.044**
KA66	−4.115 (1.946)	0.507 (0.227)	0.286	18.160	14	0.200
KA67	−0.850 (0.283)	0.717 (0.199)	0.388	36.300	25	0.067

RMSEA = 0.072, M2 = 3982.17, TLI = 0.79, CFI = 0.80. Abbreviations: S-*X^2^* = Standardized *X*^2^, RMSEA = Root Mean Square Error of Approximation, TLI = Tucker-Lewis Index, CFI = Comparative Fit Index. Items with *p*-values < 0.05 in the assessment of the item fit are highlighted in bold.

**Table 4:** Item Response Theory parameters estimate of knowledge items in complementary feeding.

Item Parameters			S-X^2^ Fit Index			
Item after Removal	Difficulty (SE)	Discrimination (SE)	Standardized Loading	X^2^	df	*p*-Value
KB1	−2.228 (0.458)	1.224 (0.331)	0.5839	10.141	8	0.255
KB2	−1.604 (0.277)	1.500 (0.355)	0.6613	9.250	9	0.415
KB3	−2.538 (0.529)	1.327 (0.390)	0.6148	9.408	5	0.094
KB4	−1.366 (0.281)	1.184 (0.282)	0.5712	14.429	11	0.210
KB5	−1.030 (0.207)	1.380 (0.311)	0.6297	19.789	11	0.048
KB6	−1.535 (0.296)	1.255 (0.300)	0.5935	13.730	9	0.132
KB7	−0.913 (0.240)	1.020 (0.250)	0.5140	24.850	12	**0.016**
KB8	−3.776 (1.501)	0.659 (0.289)	0.3610	14.892	9	0.094
KB9	1.577 (0.509)	0.717 (0.241)	0.3881	8.771	10	0.554
KB10	0.002 (0.295)	0.505 (0.188)	0.2847	6.873	13	0.909
KB11	−2.039 (1.395)	0.265 (0.177)	0.1540	13.296	14	0.503
KB12	−9.470 (10.997)	0.223 (0.264)	0.1298	17.608	9	**0.040**
KB13	3.463 (1.708)	0.468 (0.246)	0.2649	28.251	9	**0.001**
KB14	−6.935 (22.635)	0.056 (0.171)	0.0326	23.813	14	0.048
KB15	−1.297 (0.194)	2.007 (0.462)	0.7627	7.101	8	0.526
KB16	−1.587 (0.425)	0.824 (0.235)	0.4356	24.271	11	**0.012**
KB17	6.983 (7.905)	0.191 (0.211)	0.1117	9.286	10	0.505
KB18	−2.738 (0.810)	0.769 (0.263)	0.4119	7.186	9	0.618
KB19	−16.847(36.831)	-0.088 (0.212)	-0.0517	20.770	11	**0.036**
KB20	−3.165 (0.890)	1.005 (0.358)	0.5084	2.268	5	0.811
KB21	4.875 (10.536)	0.075 (0.171)	0.0439	26.505	14	**0.022**
KB22	−2.568 (1.027)	0.508 (0.212)	0.2859	16.394	12	0.174
KB23	1.068 (0.502)	0.484 (0.194)	0.2735	14.450	11	0.209
KB24	−2.055 (0.300)	2.105 (0.564)	0.7776	3.125	2	0.210
KB25	−2.222 (1.088)	0.395 (0.191)	0.2259	12.837	13	0.460
KB26	−1.126 (0.338)	0.775 (0.219)	0.4144	17.065	12	0.147
KB27	−0.669 (0.179)	1.279 (0.285)	0.6009	18.464	10	0.048
KB28	−2.016 (0.394)	1.282 (0.331)	0.6016	19.229	9	**0.023**
KB29	−2.382 (0.683)	0.775 (0.251)	0.4145	8.045	11	0.709
KB30	−4.219 (1.827)	0.679 (0.333)	0.3705	10.922	7	0.142
KB31	−1.844 (0.296)	1.690 (0.414)	0.7046	5.385	5	0.371
KB32	−2.079 (0.464)	1.071 (0.300)	0.5325	26.064	10	**0.004**
KB34	−1.690 (0.188)	3.205 (1.017)	0.8832	4.469	1	**0.035**
KB36	−0.976 (0.285)	0.860 (0.226)	0.4509	10.598	12	0.564
KB37	−2.163 (0.556)	0.876 (0.258)	0.4574	3.887	11	0.973

RMSEA = 0.064, M2 = 1019.74, TLI = 0.75, CFI = 0.77. Abbreviations: S- *X2* = Standardized *X*2, RMSEA = Root Mean Square Error of Approximation, TLI = Tucker-Lewis Index, CFI = Comparative Fit Index. Items with *p-*values < 0.05 in the assessment of the item fit are highlighted in bold.

**Table 5:** Result of factor analysis and reliability analysis of attitude in breastfeeding and formula feeding section of IYCF-CCPQ.

Factor	Item	Factor Loading λ	Communalities	Reliability ^a^
Affective-Behaviour-Cognitive	AAA18	0.479	0.229	0.89
	AAA19	0.373	0.139	
	AAA20	0.359	0.129	
	AAA21	0.474	0.225	
	AAA22	0.443	0.197	
	AAA24	0.515	0.265	
	AAA25	0.397	0.158	
	AAA27	0.440	0.193	
	AAB29	0.598	0.358	
	AAB30	0.518	0.269	
	AAB31	0.562	0.316	
	AAB32	0.675	0.455	
	AAB34	0.687	0.472	
	AAB35	0.493	0.243	
	AAB36	0.532	0.282	
	AAB37	0.316	0.099	
	AAB38	0.388	0.150	
	AAB39	0.416	0.173	
	AAB40	0.485	0.235	
	AAC1	0.480	0.230	
	AAC2	0.490	0.240	
	AAC3	0.536	0.287	
	AAC4	0.389	0.151	
	AAC5	0.486	0.236	
	AAC6	0.355	0.126	
	AAC7	0.366	0.134	
	AAC8	0.399	0.159	
	AAC9	0.561	0.315	
	AAC10	0.308	0.095	
	AAC11	0.324	0.105	
	AAC12	0.407	0.165	
	AAC14	0.515	0.265	
	AAC15	0.609	0.371	
	AAC16	0.566	0.321	

^a^ Reliability by Cronbach’s alpha.

**Table 6:** Result of factor analysis and reliability analysis of attitude in complementary feeding section of IYCF-CCPQ.

Factor	Item	Factor loading λ	Communalities	Reliability ^a^
Affective-Behavior-Cognitive	ABA25	0.40	0.158	0.90
	ABA27	0.47	0.220	
	ABA28	0.36	0.129	
	ABA30	0.60	0.358	
	ABA31	0.49	0.244	
	ABA32	0.41	0.166	
	ABA33	0.29	0.081	
	ABB34	0.20	0.038	
	ABB35	0.42	0.178	
	ABB36	0.50	0.254	
	ABB37	0.20	0.038	
	ABB38	0.50	0.254	
	ABB39	0.52	0.267	
	ABB40	0.60	0.355	
	ABB41	0.60	0.364	
	ABB42	0.55	0.300	
	ABB43	0.27	0.073	
	ABB45	0.34	0.118	
	ABB46	0.31	0.098	
	ABB48	0.57	0.324	
	ABB49	0.20	0.039	
	ABB50	0.65	0.421	
Affective-Behavior-Cognitive	ABC1	0.59	0.347	0.90
	ABC2	0.17	0.029	
	ABC3	0.52	0.271	
	ABC4	0.71	0.497	
	ABC5	0.68	0.456	
	ABC6	0.72	0.513	
	ABC7	0.36	0.127	
	ABC8	0.58	0.341	
	ABC9	0.44	0.190	
	ABC10	0.26	0.069	
	ABC11	0.48	0.227	
	ABC12	0.48	0.227	
	ABC13	0.31	0.095	
	ABC14	0.60	0.359	
	ABC16	0.15	0.022	
	ABC17	0.47	0.222	
	ABC18	0.37	0.139	
	ABC20	0.50	0.252	
	ABC22	0.43	0.182	
	ABC23	0.44	0.189	
	ABC24	0.23	0.051	

^a^ Reliability by Cronbach alpha.

**Table 7:** Descriptive statistics of the items for practices regarding breastfeeding and complementary feeding (*n* = 200).

Item	Response, *n* (%)			
	Never	Seldom	Often	Always
**A. Handling and storage of breast milk at the nursery**				
PA1. I make sure every milk storage container is labelled with the infant’s name.	15 (7.5)	4 (2.0)	5 (2.5)	176 (88.0)
PA2. I check that every expressed breast milk container that we receive has the infant’s name and the date of milk expression.	20 (10.0)	3(1.5)	9 (4.5)	168 (84.0)
PA3. I place the expressed breast milk in the refrigerator immediately upon receiving it from the parents.	19 (9.5)	7 (3.5)	4 (2.0)	170 (85.0)
PA4. I make sure that the expressed breast milk stored in the lower part of the refrigerator does not exceed 48 h.	72 (36.0)	14 (7.0)	21 (10.5)	93 (46.5)
PA5. I store again the remaining unused expressed breast milk**	134 (67.0)	25 (12.5)	6 (3.0)	35 (17.5)
PA6. I give the infants the expressed breast milk that was stored earlier first	18 (9.0)	32 (16.0)	13 (6.5)	137 (68.5)
**B. Feeding mother’s milk to the baby**				
PB1. I wash my hands with water and soap before feeding milk to an infant.	11 (5.5)	3 (1.5)	8 (4.0)	177 (89.0)
PB2. I thaw expressed breast milk in the chilled section of the refrigerator.	57(28.5)	12(6.0)	20 (10.0)	111 (55.5)
PB3. I thaw expressed breast milk by putting it in lukewarm water.	26 (13.0)	10 (5.0)	19 (9.5)	145 (72.5)
PB4. I thaw expressed breast milk in the microwave**	169 (84.5)	8 (4.0)	13 (6.5)	10 (5.0)
PB5. I discard seemingly spoiled expressed breast milk (e.g., smells sour, discolored)	29 (14.5)	34 (17.0)	14 (7.0)	121 (61.5)
PB6. I give expressed breast milk at an appropriate temperature.	25 (12.5)	9 (4.5)	16 (8.0)	150 (75.0)
PB7. I give expressed breast milk within an hour after thawing.	58 (29.0)	27 (13.5)	17 (8.5)	98 (49.0)
PB8. I shake expressed breast milk before giving it to an infant**	57 (28.5)	11 (5.5)	14 (7.0)	118 (59.0)
PB9. I discard the remaining expressed breast milk if it is not completely consumed.	45 (22.5)	32 (16.0)	18 (9.0)	105 (52.5)
Statement**=reverse statement				
PB10. I give expressed breast milk according to the infant’s demand.	36 (18.0)	15 (7.5)	23 (11.5)	126 (63.0)
PB11. I give expressed breast milk to the infant using a cup/spoon/syringe.	142 (71.0)	25 (12.5)	9 (4.5)	24 (12.0)
PB12. I burp the infant after a breast milk feeding.	19 (9.5)	9 (4.5)	12 (6.0)	160 (80.0)
PB13. I give plain water after a breast milk feeding.**	105 (52.5)	31 (15.5)	19 (9.5)	45 (22.5)
**C. Preparation and handling of formula milk**				
PC1. I clean the kitchen surfaces with soap before preparing the formula	32 (16.0)	16 (8.0)	58 (29.0)	94 (47.0)
PC2. I wash my hands with water and soap before preparing the formula	3 (1.5)	3 (1.5)	12 (6.0)	182 (91.0)
PC3. I cook the water until it is boiling.	2 (1.0)	3 (1.5)	9 (4.5)	186 (93.0)
PC4. I prepare the formula according to the instructions given on the formula label.	1 (0.5)	4 (2.0)	7 (3.5)	188 (94.0)
PC5. I prepare the formula with water at a temperature of 70°C.	32 (16.0)	16 (8.0)	19 (9.5)	133 (66.5)
PC6. I add the formula powder in the right quantity.	6 (3.0)	3 (1.5)	10 (5.0)	181 (90.5)
PC7. I shake the milk bottle with its cap in place to make sure that the milk is well-mixed.	3 (1.5)	3 (1.5)	13 (6.5)	181 (90.5)
PC8. I put the milk bottle under running tap water to lower its temperature.	58 (29.0)	32 (16.0)	26 (13.0)	84 (42.0)
**D. Handling and cleaning of milk bottle**				
PD1. I sterilize the milk bottle before use.	10 (5.0)	8 (4.0)	52 (26.0)	130 (65.0)
PD2. I place the milk bottle in boiling water for sterilization	31 (15.5)	27 (13.5)	79 (39.5)	63 (31.5)
PD3. I keep sterilized milk bottles in a closed container.	14 (7.0)	13 (6.5)	28 (14.0)	145 (72.5)
PD4. I wash the milk bottles using a soft sponge.	4 (2.0)	7 (3.5)	14 (7.0)	175 (87.5)
PD5. I wash the milk bottles using soap.	34 (17.0)	25 (12.5)	22 (11.0)	119 (59.5)
**E. Preparing, handling and storing food**				
PE1. I wash my hands with water and soap before preparing food.	1 (0.5)	2 (1.0)	8 (4.0)	189 (94.5)
PE2. I make sure the children’s hands are washed with water and soap before they eat.	1 (0.5)	9 (4.5)	11 (5.5)	178 (89.5)
PE3. I make sure that the plates, cups, spoons, and forks that are to be used are clean.	2 (1.0)	2 (1.0)	5 (2.5)	190 (95.5)
Statement **= reverse statement				
PE4. I make sure the plates and cups that are to be used are not cracked or scratched.	4 (2.0)	3 (1.5)	4 (2.0)	189 (94.5)
PE5. I make sure that the cooked food kept at room temperature is given to the children within 2 h.	42 (21.0)	17 (8.5)	23 (11.5)	118 (59.0)
**F. Responsive feeding**				
PF1. I interact with the children during the meal	4 (2.0)	21 (10.5)	37 (18.5)	138 (69.0)
PF2. I encourage the children to feed themselves.	23 (11.5)	24 (12.0)	29 (14.5)	121 (62.0)
PF3. I feed the children when there are signs of hunger only**	86 (43.0)	18 (9.0)	25 (12.5)	71 (35.5)
PF4. I give the children time to finish their food.	4 (2.0)	8 (4.0)	16 (8.0)	171 (86.0)
PF5. I do not scold or penalize the children if they refuse to eat.	62 (31.0)	19 (9.5)	10 (5.0)	109 (54.5)

Statement ** = reverse statement.

**Table 8:** Summary of items in IYCF-CCPQ before and after psychometric analyses.

Domain	Before		After	
	Section	Item	Domain	Item
Knowledge	Breastfeeding and formula feeding	67 (27 reverse-scored items)	Breastfeeding and formula feeding	64 (26 reverse-score items)
	Complementary feeding	37 (20 reverse-scored items)	Complementary feeding	35 (15 reverse-scored items)
Attitude	Breastfeeding and formula feeding	40 (12 reverse-scored items)	Breastfeeding and formula feeding	34 (9 reverse-scored items)
	Complementary feeding	50 (23 reverse scored items)	Complementary feeding	43 (18 reverse scored items)
Practices	Handling EBM	6 (1 negative statement)	Handling EBM	6 (1 negative statement)
	Giving EBM	13 (3 negative statement)	Giving EBM:	13 (3 negative statement)
	Handling formula milk	8 items	Handling formula milk:	8 items
	Handling feeding bottle	5 items	Handling milk bottle:	5 items
	Food Storage	5 items	Food Storage:	5 items
	Responsive feeding	3 items (1 negative statement)	Responsive feeding	4 items (1 negative statement)

EBM: expression of breast milk.

## Supplementary Materials

The following are available online at https://www.mdpi.com/1660-4601/16/12/2147/s1. The IYCF-CCPQ questionnaire (KAP section) in English version.
