# Supplementary material for: Validation of Infant and Young Child Feeding Questionnaire for the Assessment of Knowledge, Attitudes and Practices among Child Care Providers: The IYCF-CCPQ
Source: Int J Environ Res Public Health. 2019 Jun 17;16(12):2147. doi: 10.3390/ijerph16122147 (PMC6617158; doi:10.3390/ijerph16122147)
Supplement: Supplementary file 1 [file ijerph-16-02147-s001.pdf]

KNOWLEDGE

Check (✓) your answer choice. Answer all of the questions.

SECTION A: BREASTFEEDING AND INFANT FORMULA

	True	False	Do not know
1. Colostrum has a high antibody content.	==☐==	☐	☐
2. Breast milk causes constipation in infants.	☐	==☐==	☐
3. Breastfeeding reduces the risk of infants getting lung infections.	==☐==	☐	☐
4. Breast milk that looks diluted is less nutritious.	☐	==☐==	☐
5. Breastfeeding causes infants to have low body weights.	☐	==☐==	☐
6. The common cold risk can be reduced by breast milk.	==☐==	☐	☐
7. Breast milk is not easily contaminated when compared to formula.	==☐==	☐	☐
8. Breastfeeding calms the infant.	==☐==	☐	☐
9. Exclusive breastfeeding can space pregnancies.	==☐==	☐	☐
10. Mothers who breastfeed are at a higher risk of getting breast cancer.	☐	==☐==	☐
11. Breastfeeding infants have daily bowel movements.	==☐==	☐	☐
12. Infants will have normal weight gain with optimal breastfeeding.	==☐==	☐	☐
13. Breastfeeding increases an infant’s intelligence.	==☐==	☐	☐
14. Infants sleep well when they are getting sufficient breast milk.	==☐==	☐	☐
15. Breastfeeding causes infant to have diarrhea.	☐	==☐==	☐
16. Breastfeeding reduces the incidence of child abuse.	==☐==	☐	☐
17. Breastfeeding causes maldevelopment of the gums and teeth.	☐	==☐==	☐
18. Colostrum is difficult to digest.	☐	==☐==	☐
19. Breastfeeding should continue even when the child is sick.	==☐==	☐	☐

	True	False	Do not know
20. Formula provides better protection from allergies when compared to breast milk.	☐	==☐==	☐
21. Infants should be given only breast milk from birth until 6 months of age.	==☐==	☐	☐
22. Breastfeeding should be in accordance with the infant’s demand.	==☐==	☐	☐
23. The use of artificial teats interferes with tooth development.	==☐==	☐	☐
24. Infants can be given plain water during the exclusive breastfeeding period.	☐	==☐==	☐
25. Exclusive breastfeeding means that infants are only given breast milk from birth to 6 months of age.	==☐==	☐	☐
26. Colostrum can cause infants to have difficulty defecating.	☐	==☐==	☐
27. Breastfeeding should be continued until the infant is 2 years old or older.	==☐==	☐	☐
28. The mother has to breastfeed every 3 hours.	☐	==☐==	☐
29. The correct position during breastfeeding ensures effective breastfeeding.	==☐==	☐	☐
30. Expressed breast milk that has been thawed can be stored again.	☐	==☐==	☐
31. The expressed breast milk container needs to be labelled with infant’s name, time, and date.	==☐==	☐	☐
32. Frozen expressed breast milk can be thawed on the stove.	☐	==☐==	☐
33. Breastfeeding should be continued up to 2 years or more in addition to complementary food.	==☐==	☐	☐
34. The use of an artificial teat facilitates breastfeeding.	☐	==☐==	☐
35. Expressed breast milk can be stored for up to 3 months in the freezer of a 2-door refrigerator.	==☐==	☐	☐
36. Providing plain water after each breastfeeding session is encouraged.	☐	==☐==	☐
37. Newly expressed breast milk can be mixed together in a container with previously expressed breast milk.	☐	==☐==	☐
38. Formula is nutritious because it contains added docosahexaenoic acid (DHA).	☐	==☐==	☐

	True	False	Do not know
39. Frozen expressed breast milk can be thawed in the microwave.	☐	**☐**	☐
40. Expressed breast milk that has been thawed completely should be consumed in an hour.	**☐**	☐	☐
41. Formula can be kept longer at room temperature when compared to breast milk.	☐	**☐**	☐
42. Frozen expressed breast milk that has been thawed can be stored in the chilled section of the refrigerator for 24 hours.	**☐**	☐	☐
43. Expressed breast milk stored in the chilled section of the refrigerator needs to be consumed immediately if the power supply is disconnected.	**☐**	☐	☐
44. Frozen expressed breast milk can be thawed in lukewarm water.	**☐**	☐	☐
45. Formula is a kind of complementary food.	☐	**☐**	☐
46. The "first in, first out" method refers to expressed breast milk that has been stored earlier being consumed first.	**☐**	☐	☐
47. Frozen expressed breast milk that has been thawed can be refrozen again.	☐	**☐**	☐
48. The preparation of formula should follow the guidelines provided.	**☐**	☐	☐
49. Expressed breast milk can last for 4 hours at room temperature.	**☐**	☐	☐
50. Formula has high quality protein.	☐	**☐**	☐
51. The formula powder should be put in the bottle first, before adding the water.	☐	**☐**	☐
52. Boiling water can be poured directly into the milk bottle when preparing the formula.	☐	**☐**	☐
53. The temperature of the milk should be checked by putting a few drops of it onto the carer's wrist.	**☐**	☐	☐
54. Formula should be prepared with water heated to 70°C.	**☐**	☐	☐
55. Formula is not easily contaminated.	☐	**☐**	☐
56. The outside of the milk bottle should be dried before giving it to the infant.	**☐**	☐	☐

	True	False	Do not know
57. After preparing the formula, the bottle should be placed under running tap water to lower the milk temperature.	☐	☐	☐
58. Milk bottles should be sterilized once a week.	☐	☐	☐
59. Bisphenol A (BPA)-free milk bottles are safe for use.	☐	☐	☐
60. Sterilized bottles can be stored in open spaces.	☐	☐	☐
61. Milk bottles need to be boiled in water for proper sterilization.	☐	☐	☐
62. Milk bottles need to be replaced every 6 months if they are not scratched.	☐	☐	☐
63. Milk bottles can be cleansed using a soft sponge.	☐	☐	☐
64. Milk bottles can be sterilized using "steam sterilization."	☐	☐	☐

SECTION B: COMPLEMENTARY FOOD FOR INFANTS AND CHILDREN

	True	False	Do not know
1. Complementary food needs to be introduced when the infant is 6 months old.	☐	☐	☐
2. A balanced diet reduces the risk of skin disease (eczema) in children.	☐	☐	☐
3. A child's daily diet should follow the food pyramid.	☐	☐	☐
4. Scaly skin problems can occur in children who do not receive a balanced diet.	☐	☐	☐
5. Complementary feeding can be started at 4 months of age.	☐	☐	☐
6. After 6 months, breast milk alone is insufficient to provide all of an infant's nutritional needs.	☐	☐	☐
7. Fruits can be given to a 7-month-old infant.	☐	☐	☐
8. Tea/coffee can be given to children.	☐	☐	☐
9. One teaspoon of fat/oil should be added to an infant's daily meals.	☐	☐	☐
10. At the age of 1 year, food needs to be mashed.	☐	☐	☐
11. Family food can be given to a 9-month-old infant.	☐	☐	☐

	True	False	Do not know
12. Canned drinks can be given to children.	☐	==☐==	☐
13. A soft diet should be continued for infants aged 12 months and older.	☐	==☐==	☐
14. Prawns can be given to a 10-month-old infant.	☐	==☐==	☐
15. Try different combinations of foods, textures, and flavors if the children refuse to eat.	==☐==	☐	☐
16. Finger food can be given to a 9-month-old infant.	==☐==	☐	☐
17. Honey can be given to an 8-month-old infant.	☐	==☐==	☐
18. A soft diet needs to be given when the child is sick.	==☐==	☐	☐
19. Eggs can be given to a 9-month-old infant.	☐	==☐==	☐
20. Carers need to accompany children when they are eating nuts.	==☐==	☐	☐
21. A 9-month-old infant is allowed to hold food and feed him or herself.	==☐==	☐	☐
22. Whole nuts can be given to a 10-month-old infant.	☐	==☐==	☐
23. The food pyramid has 3 main levels.	☐	==☐==	☐
24. The rice and grain group provides energy for children.	==☐==	☐	☐
25. Foods in the vegetable group need to be consumed more often than those in the rice and grain group.	==☐==	☐	☐
26. Cooked food should not be left at room temperature for longer than 2 hours.	==☐==	☐	☐
27. Give food more frequently when a child is recovering from an illness.	==☐==	☐	☐
28. Interacting with children during meal times can encourage them to eat.	==☐==	☐	☐
29. The same cutting board can be used for cutting raw meat and fruits.	☐	==☐==	☐
30. A cracked plate can be used to prepare food for children.	☐	==☐==	☐
31. Encourage children to drink plenty of water when they are sick.	==☐==	☐	☐
32. Children need to be penalized if they refuse to eat.	☐	==☐==	☐

	True	False	Do not know
33. Different methods of encouragement should be attempted if a child refuses to eat.	☐	☐	☐
34. Eating while watching television helps children to eat.	☐	☐	☐
35. Children should be encouraged to feed themselves their own food.	☐	☐	☐

ATTITUDE

Answer all of the questions according to the scale below. Check (✓) the box with your answer choice.

<u>SECTION A: BREASTFEEDING AND INFANT FORMULA</u>

1<br>Strongly disagree	2<br>Disagree	3<br>Not sure	4<br>Agree	5<br>Strongly agree

	1	2	3	4	5
1. I believe mother's milk is important for an infant.	☐	☐	☐	☐	☐
2. I think infants who drink mother's milk are easier to manage.	☐	☐	☐	☐	☐
3. I believe that breast milk is economical.	☐	☐	☐	☐	☐
4. I assume breastfeeding requires expensive equipment.	☐	☐	☐	☐	☐
5. I think breastfeeding is something outdated.	☐	☐	☐	☐	☐
6. I think expressed breast milk is easier to manage than formula.	☐	☐	☐	☐	☐
7. I think exclusive breastfeeding is the best option for working mothers.	☐	☐	☐	☐	☐
8. I think breast milk increases the bond between mothers and infants.	☐	☐	☐	☐	☐
9. I think breastfeeding is tiring.	☐	☐	☐	☐	☐
10. I think infants who drink formula choke more easily.	☐	☐	☐	☐	☐
11. I think taking care of a breastfed infant is more difficult than taking care of those who drink formula.	☐	☐	☐	☐	☐
12. I believe breast milk is nutritious.	☐	☐	☐	☐	☐
13. I think breastfeeding is a waste of time.	☐	☐	☐	☐	☐
14. I believe infants who drink breast milk are healthier.	☐	☐	☐	☐	☐
15. I think breastfeeding is embarrassing.	☐	☐	☐	☐	☐
16. I am proud to see working mothers continue breastfeeding.	☐	☐	☐	☐	☐

1	2	3	4	5
**Strongly disagree**	**Disagree**	**Not sure**	**Agree**	**Strongly agree**

	1	2	3	4	5
17. I feel that it is easy to handle expressed breast milk.	☐	☐	☐	☐	==☐==
18. I do not like seeing mothers spend time on breastfeeding.	==☐==	☐	☐	☐	☐
19. I am happy that breastfed infants grow well.	☐	☐	☐	☐	==☐==
20. I am happy that breastfed infants rarely have fevers.	☐	☐	☐	☐	==☐==
21. I am proud to see mothers who breastfeed.	☐	☐	☐	☐	==☐==
22. I am embarrassed to see mothers breastfeeding in this childcare center.	==☐==	☐	☐	☐	☐
23. I enjoy taking care of breastfed infants.	☐	☐	☐	☐	==☐==
24. I will support the practice of breastfeeding in childcare centers.	☐	☐	☐	☐	==☐==
25. I will encourage mothers to breastfeed in this childcare center.	☐	☐	☐	☐	==☐==
26. I will recommend that mothers join the breastfeeding support group.	☐	☐	☐	☐	==☐==
27. I will increase my knowledge of breastfeeding.	☐	☐	☐	☐	==☐==
28. I will handle expressed breast milk with the utmost care.	☐	☐	☐	☐	==☐==
29. I will participate in the breastfeeding training program.	☐	☐	☐	☐	==☐==
30. I will receive the expressed breast milk to be used in this childcare center.	☐	☐	☐	☐	==☐==
31. I will encourage mothers to give formula as well as breast milk if the infant is 6 months old.	==☐==	☐	☐	☐	☐
32. I will make sure the infant is burped sufficiently after a milk feeding.	☐	☐	☐	☐	==☐==
33. I will correct any inaccurate information regarding breastfeeding.	☐	☐	☐	☐	==☐==
34. I will disseminate information on the benefits of breastfeeding.	☐	☐	☐	☐	==☐==

SECTION B: PROVIDING COMPLEMENTARY FEEDING

1	2	3	4	5
Strongly disagree	Disagree	Not sure	Agree	Strongly agree

	1	2	3	4	5
1. I believe complementary food is important.	☐	☐	☐	☐	**☐**
2. I think children should be given a variety of foods.	☐	☐	☐	☐	**☐**
3. I think the food preparation for the children is tedious.	**☐**	☐	☐	☐	☐
4. I think the food texture should be gradually changed according to the child's age.	☐	☐	☐	☐	**☐**
5. I think breastfeeding should be continued in addition to complementary food.	☐	☐	☐	☐	**☐**
6. I think complementary food should be given in the correct quantity.	☐	☐	☐	☐	**☐**
7. I think the feeding frequency should be increased according to the child's age.	☐	☐	☐	☐	**☐**
8. I believe children should be encouraged when they are feeding.	☐	☐	☐	☐	**☐**
9. I believe children should take in more water when they are sick.	☐	☐	☐	☐	**☐**
10. I assume instant infant food is sufficient for the children during the day.	**☐**	☐	☐	☐	☐
11. I think complementary food should be introduced at an appropriate age.	☐	☐	☐	☐	**☐**
12. I think a banana is suitable for a 3-month-old infant.	**☐**	☐	☐	☐	☐
13. I think breastfeeding can be discontinued after the introduction of complementary food.	**☐**	☐	☐	☐	☐
14. I believe interacting with children is important during feeding.	☐	☐	☐	☐	**☐**
15. I believe children are healthier if complementary food is introduced at the age of 4 months.	**☐**	☐	☐	☐	☐
16. I believe children are healthy when they have adequate complementary food.	☐	☐	☐	☐	**☐**
17. I believe tea is good for a child's health.	**☐**	☐	☐	☐	☐

1	2	3	4	5
**Strongly disagree**	**Disagree**	**Not sure**	**Agree**	**Strongly agree**

	**1**	**2**	**3**	**4**	**5**
18. I think instant infant food is of a better quality than home cooked food.	==☐==	☐	☐	☐	☐
19. I think vegetables are not suitable for children.	==☐==	☐	☐	☐	☐
20. I think it is appropriate for children to have their meals in front of the television.	==☐==	☐	☐	☐	☐
21. I think instant infant food is good for children.	==☐==	☐	☐	☐	☐
22. I am happy to prepare a variety of foods for the children.	☐	☐	☐	☐	==☐==
23. I am happy to interact with the children during a meal.	☐	☐	☐	☐	==☐==
24. I am happy seeing children eating in front of the television.	==☐==	☐	☐	☐	☐
25. I am tired of preparing the children’s meals.	==☐==	☐	☐	☐	☐
26. I do not like giving vegetables to children.	==☐==	☐	☐	☐	☐
27. I am happy to cook food for the children.	☐	☐	☐	☐	==☐==
28. I am comfortable using instant infant food.	==☐==	☐	☐	☐	☐
29. I will let the children eat whatever they like.	==☐==	☐	☐	☐	☐
30. I will discuss the children’s nutritional plan with the childcare center supervisor.	☐	☐	☐	☐	==☐==
31. I will interact with the children during their meals.	☐	☐	☐	☐	==☐==
32. I will give the same food to all of the infants and children.	==☐==	☐	☐	☐	☐
33. I will provide time for the children to eat.	☐	☐	☐	☐	==☐==
34. I will give encouragement for the children to eat.	☐	☐	☐	☐	==☐==
35. I will try a combination or a variety of foods if the children refuse to eat.	☐	☐	☐	☐	==☐==
36. I will give the children a soft diet (e.g., congee) when they are sick.	☐	☐	☐	☐	==☐==
37. I will encourage the children to drink more water when they are sick.	☐	☐	☐	☐	==☐==

1	2	3	4	5
**Strongly disagree**	**Disagree**	**Not sure**	**Agree**	**Strongly agree**

	**1**	**2**	**3**	**4**	**5**
38. I will give frequent meals to children recovering from illnesses.	☐	☐	☐	☐	☐
39. I will penalize the children for refusing to eat.	☐	☐	☐	☐	☐
40. I will encourage the children to eat while watching television.	☐	☐	☐	☐	☐
41. I will use different methods of encouragement if a child refuses to eat.	☐	☐	☐	☐	☐
42. I will encourage a child to feed him/herself.	☐	☐	☐	☐	☐
43. I will give different textures of food according to a child’s age.	☐	☐	☐	☐	☐

PRACTICE

Answer all of the questions according to the scale below. Check (✓) your choice of answer.

<u>BREASTFEEDING, INFANT FORMULA, AND COMPLEMENTARY FOOD</u>

1	2	3	4
**Never**<br>*(never do)*	**Seldom**<br>*(once a month)*	**Frequent**<br>*(2-3 days per week)*	**Always**<br>*(everyday)*

A. Handling and storing breast milk at the childcare center

		1	2	3	4
1.	I make sure every milk storage container is labelled with the infant's name.	☐	☐	☐	**☐**
2.	I check that every expressed breast milk container that we receive has the infant's name and the date of milk expression.	☐	☐	☐	**☐**
3.	I place the expressed breast milk in the refrigerator immediately upon receiving it from the parents.	☐	☐	☐	**☐**
4.	I make sure that the expressed breast milk stored in the lower part of the refrigerator does not exceed 48 hours.	☐	☐	☐	**☐**
5.	I store again the remaining unused expressed breast milk.	**☐**	☐	☐	☐
6.	I give the infants the expressed breast milk that was stored earlier first.	☐	☐	☐	**☐**

B. Giving mother's milk to the infant

		1	2	3	4
1.	I wash my hands with water and soap before feeding milk to an infant.	☐	☐	☐	**☐**
2.	I thaw expressed breast milk in the chilled section of the refrigerator.	☐	☐	☐	**☐**
3.	I thaw expressed breast milk by putting it in lukewarm water.	☐	☐	☐	**☐**
4.	I thaw expressed breast milk in the microwave.	**☐**	☐	☐	☐
5.	I discard seemingly spoiled expressed breast milk (e.g., smells sour, discolored).	☐	☐	☐	**☐**

1	2	3	4
**Never**<br>*(never do)*	**Seldom**<br>*(once a month)*	**Frequent**<br>*(2-3 days per week)*	**Always**<br>*(everyday)*

	1	2	3	4
6. I give expressed breast milk at an appropriate temperature.	☐	☐	☐	==☐==
7. I give expressed breast milk within an hour after thawing.	☐	☐	☐	==☐==
8. I shake expressed breast milk before giving it to an infant.	☐	☐	☐	==☐==
9. I discard the remaining expressed breast milk if it is not completely consumed.	☐	☐	☐	==☐==
10. I give expressed breast milk according to the infant's demand.	☐	☐	☐	==☐==
11. I give expressed breast milk to the infant using a cup/spoon/syringe.	☐	☐	☐	==☐==
12. I burp the infant after a breast milk feeding.	☐	☐	☐	==☐==
13. I give plain water after a breast milk feeding.	==☐==	☐	☐	☐

C. Preparation and handling of the infant formula

	1	2	3	4
1. I clean the kitchen surfaces with soap before preparing the formula.	☐	☐	☐	==☐==
2. I wash my hands with water and soap before preparing the formula.	☐	☐	☐	==☐==
3. I cook the water until it is boiling.	☐	☐	☐	==☐==
4. I prepare the formula according to the instructions given on the formula label.	☐	☐	☐	==☐==
5. I prepare the formula with water at a temperature of 70°C.	☐	☐	☐	==☐==
6. I add the formula powder in the right quantity.	☐	☐	☐	==☐==
7. I shake the milk bottle with its cap in place to make sure that the milk is well-mixed.	☐	☐	☐	==☐==
8. I put the milk bottle under running tap water to lower its temperature.	☐	☐	☐	==☐==

1	2	3	4
**Never** *(never do)*	**Seldom** *(once a month)*	**Frequent** *(2-3 days per week)*	**Always** *(everyday)*

D. Handling and cleaning the milk bottle

		1	2	3	4
1.	I sterilize the milk bottle before use.	☐	☐	☐	==☐==
2.	I place the milk bottle in boiling water for sterilization.	☐	☐	☐	==☐==
3.	I keep sterilized milk bottles in a closed container.	☐	☐	☐	==☐==
4.	I wash the milk bottles using a soft sponge.	☐	☐	☐	==☐==
5.	I wash the milk bottles using soap.	☐	☐	☐	==☐==

E. Preparing, handling, and storing food

		1	2	3	4
1.	I wash my hands with water and soap before preparing food.	☐	☐	☐	==☐==
2.	I make sure the children’s hands are washed with water and soap before they eat.	☐	☐	☐	==☐==
3.	I make sure that the plates, cups, spoons, and forks that are to be used are clean.	☐	☐	☐	==☐==
4.	I make sure the plates and cups that are to be used are not cracked or scratched.	☐	☐	☐	==☐==
5.	I make sure that the cooked food kept at room temperature is given to the children within 2 hours.	☐	☐	☐	==☐==

F. Responsive feeding

		1	2	3	4
1.	I interact with the children during the meal.	☐	☐	☐	==☐==
2.	I encourage the children to feed themselves.	☐	☐	☐	==☐==
3.	I feed the children when there are signs of hunger only.	==☐==	☐	☐	☐
4.	I give the children time to finish their food.	☐	☐	☐	==☐==
5.	I do not scold or penalize the children if they refuse to eat.	☐	☐	☐	==☐==
